# Breaking New Ground in Interventional Pulmonology: Integrating Cone Beam CT and Robotic-Assisted Bronchoscopy for High-Risk Pneumothorax in Peripherally Located Solitary Pulmonary Nodules

**DOI:** 10.7759/cureus.62532

**Published:** 2024-06-17

**Authors:** Amir R Reihani, Mahshid Zohouri, Justin Thomas

**Affiliations:** 1 Pulmonary and Critical Care Medicine, Eisenhower Medical Center, Rancho Mirage, USA; 2 Critical Care Medicine, Stony Brook University, Stony Brook, USA; 3 Internal Medicine, Eisenhower Medical Center, Rancho Mirage, USA; 4 Interventional Pulmonary and Critical Care Medicine, Eisenhower Medical Center/University of California Riverside, Rancho Mirage, USA

**Keywords:** high-risk pneumothorax, radial endobronchial ultrasound (r-ebus), rapid on-site evaluation (rose), robotic-assisted bronchoscopy (rab), peripheral pulmonary lesions (ppl), cone-beam computed tomography (cbct), lung cancer

## Abstract

Lung cancer, a leading cause of global cancer-related deaths, necessitates the development of innovative diagnostic techniques. Traditional bronchoscopy, while useful, has limitations in diagnosing peripheral pulmonary lesions (PPLs) and carries a higher risk of complications such as pneumothorax. However, the field of interventional pulmonology has seen significant advancements, including the introduction of robotic-assisted bronchoscopy (RAB), cone-beam computed tomography (CBCT), radial endobronchial ultrasound (R-EBUS), and rapid on-site evaluation (ROSE). These advancements have greatly improved the precision of diagnosing high-risk PPLs. This report presents the case of a 60-year-old female smoker with chronic obstructive pulmonary disease and extensive centrilobular emphysema, who had a peripherally located high-risk pulmonary nodule. She was successfully diagnosed with metastatic adenocarcinoma using an integrated approach, despite the challenging location of the lesion and high risk of pneumothorax. The integration of RAB with CBCT and augmented fluoroscopy offers a groundbreaking approach for diagnosing and managing difficult-to-reach, high-risk pulmonary nodules, marking a significant stride in the field of interventional pulmonology.

## Introduction

Lung cancer is the most common cause of cancer-related deaths in both genders globally [1], necessitating innovations in diagnostic techniques. Historically, the role of bronchoscopy in obtaining adequate biopsies from peripheral pulmonary lesions (PPLs) was limited due to its constrained diagnostic precision and higher risks for complications, including pneumothorax in emphysematous patients [2]. However, advances in interventional pulmonology with the integration of robotic-assisted bronchoscopy (RAB) have revolutionized this process [3], allowing practitioners to reach PPLs beyond the airways with improved accuracy, thereby enhancing diagnostic yield.

Nevertheless, precise lesion localization during the procedure can present challenges. This case report demonstrates a pioneering application of combined cone beam computed tomography (CBCT) and RAB with radial endobronchial ultrasound (R-EBUS) and rapid on-site cytologic evaluation (ROSE) by a cytotechnician. This approach elevates the procedure's effectiveness, particularly in cases of PPLs at high risk for malignancy and procedural pneumothorax [4].

Our case underscores the importance of multiple imaging modalities to improve precise lesion localization. It demonstrates how these modalities can be combined to achieve a high diagnostic yield, even in challenging cases. For instance, stricture preventing robotic bronchoscope advancement required balloon bronchoplasty to dilate the airway, allowing for closer navigation to the lesion. The integration of CBCT and augmented fluoroscopy provided real-time 3D visualization, which is crucial for accurate tool placement and minimizing complications [5,6]. 

This report highlights the convergence of state-of-the-art interventional technologies, showcasing refined biopsy techniques for difficult-to-reach lesions in high-risk locations. Our findings align with recent studies, including a meta-analysis published in the Annals of the American Thoracic Society, which reported increased diagnostic yield and safety using RAB and advanced imaging techniques [7]. By discussing similar cases recently published [8,9], our report contributes to the growing body of evidence supporting these integrated techniques as a standard of care for diagnosing high-risk peripheral pulmonary lesions.

In summary, this case emphasizes the critical role of advanced interventional pulmonology in managing PPLs among patients with higher risks of procedural complications, providing a template for clinicians facing similar diagnostic challenges.

## Case presentation

A 60-year-old female presented to the interventional pulmonary clinic for evaluation of a lung nodule seen on CT of the chest done for staging workup of the recently diagnosed colon cancer. She was recently diagnosed with invasive poorly differentiated adenocarcinoma of the colon, T4aN1Mx, status-post colectomy and small-bowel resection. She had a 20-pack-year history of smoking, a moderate chronic obstructive pulmonary disease with significant centrilobular and paraseptal emphysema, and a family history of lung cancer.

**Figure 1:**
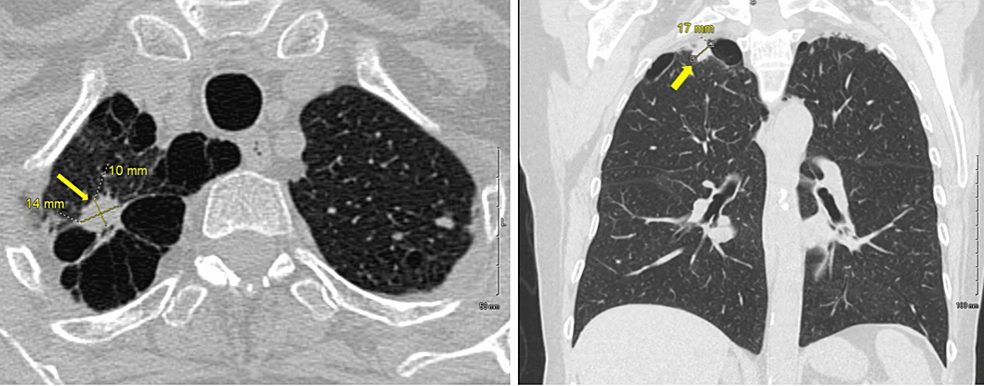
Right apical, spiculated, subpleural nodule, measuring 17 x 14 x 10 mm (yellow arrows) with surrounding severe para septal emphysema

Initial chest CT without contrast revealed a 17x14x10 mm spiculated right upper lobe, apical segment pulmonary nodule (Figure 1). 

**Figure 2:**
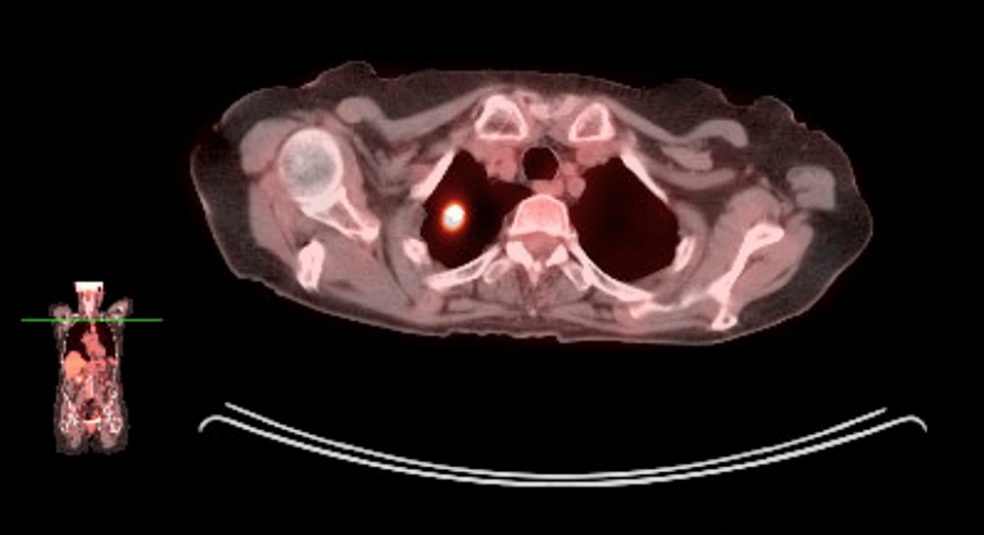
Whole body PET scan utilizing 16.831 mCi of F18-FDG demonstrates a 1.7 cm noncalcified, solid, speculated nodule in the right apical lung region, exhibiting high-grade metabolic activity with a maximum SUV of 12.9, set against a backdrop of surrounding pulmonary emphysema. SUV:  standardized uptake value; FDG: fluorodeoxyglucose

A whole-body PET scan was done showing high-grade activity in the pulmonary nodule. Although a metastasis from colon cancer was possible, the lesion’s appearance and the patient’s risk factors raised concern for a primary lung carcinoma (Figure 2).

**Figure 3:**
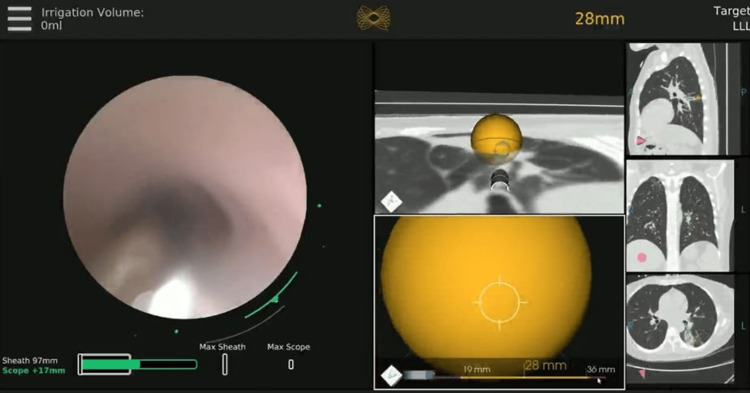
Robotic-assisted bronchoscopy for first-pass sampling of peripherally located lung nodule Image on the left side displays the real-time scope view with the ability to see our instruments in the periphery to ensure sample quality in the right upper lobe apical subsegmental bronchus.  Middle images show the generated pathway to the right upper lobe nodule and display targets straight ahead. CT images on the right side show the corresponding sagittal, coronal, and axial views of the tumor in the right upper lobe.

The patient underwent RAB (MONARCH™; Ethicon, Inc., Raritan, New Jersey, United States) with R-EBUS (Olympus Corporation, Shinjuku City, Tokyo, Japan) guidance to meticulously locate the precise location of the right upper lobe of the lung. Although the proper airway was located by electromagnetic navigation, due to the stricture of the airway, the robotic bronchoscope could not be advanced closer than 35 mm to the lesion (Figure 3).

**Figure 4:**
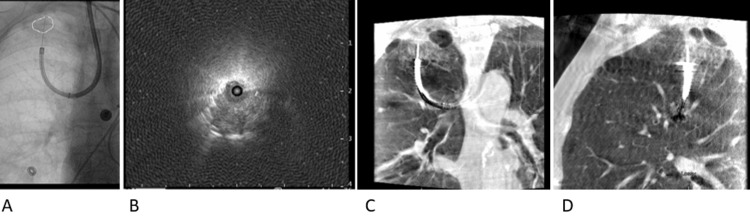
Augmented fluoroscopy to guide needle placement, brush placement, and transbronchial biopsies (A); Eccentric R-EBUS view of the lesion (B); Cone-beam CT coronal images (C) and sagittal images (D) were used to confirm the needle placement on the first pass.

Multiple passes with fine needle aspiration (FNA) were performed using augmented fluoroscopy with three-dimensional (3D) rendering of the lesion (Figure 4A). A 4x20 mm Charger^TM^ over-the-wire PTA balloon dilatation catheter (Boston Scientific Corporation, Marlborough, Massachusetts, United States) was inserted through the working channel of the RAB and directed into the airway of interest. The balloon was slowly dilated to 4 mm, and the robotic scope was advanced closer to the lesion using the Seldinger technique to gain access to the lesion. A 21G Periview Flex needle (Olympus Corporation) was inserted through the RAB to the target lesion using fluoroscopic guidance followed immediately by a spin of the CBCT (ARTIS pheno; Siemens AG, Munich, Germany) confirming the needle in the lesion (Figures 4C, 4D). Augmented 3D images were used during the needle and brush placement, as well as during the opening and closing of the forceps to help prevent excessive distal placement of the tools and prevent pneumothorax (Figures 4C, 4D).

**Figure 5:**
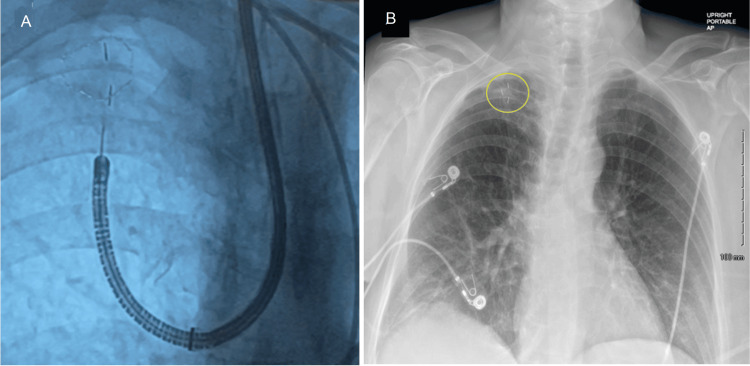
(A) Visicoil gold 5x0.5 mm fiducial markers placed using augmented fluoroscopy; (B) Post-procedural chest radiograph showing three fiducial markers placed in a triangulated pattern around the lesion and no evidence of pneumothorax

Following this, three 0.50 mm x 0.5 cm gold fiducial markers (Visicoil, IZI Medical Products, Maryland, United States) were placed in a triangulated pattern around the lesion using electromagnetic navigation (EMN) as well as augmented fluoroscopy to assist with possible stereotactic body radiation therapy of this small peripheral lung lesion (Figure 5).

**Figure 6:**
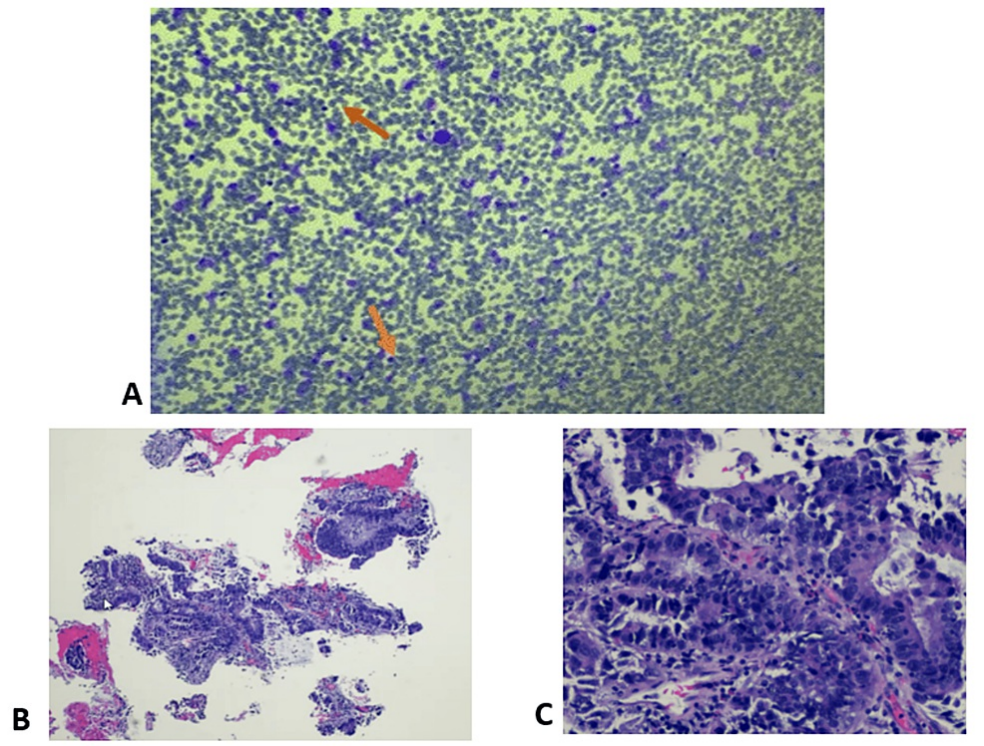
Histopathological evaluation of transbronchial biopsy from the right upper lobe. A: H&E staining demonstrates clusters of malignant columnar cells (orange arrows) amidst histiocytes and blood, with ROSE assisting the evaluation. B: H&E staining at original magnification of x100. C: Higher magnification at x200 reveals features characteristic of adenocarcinoma. ROSE: rapid on-site evaluation

After ROSE confirmed malignant cells, the RAB was removed, and the curvilinear EBUS scope was used for mediastinal and hilar lymph node evaluation. Multiple transbronchial needle aspirations (TBNA) were performed at stations 11L, 10L, 4L, 7, and 11R. Final pathology and cytology results from right upper lobe transbronchial biopsies and TBNA revealed metastatic adenocarcinoma consistent with colorectal origin (Figure 6) with negative mediastinal and hilar lymph nodes.

The patient tolerated the procedure well and was discharged the same day in stable condition without postprocedural pneumothorax.

## Discussion

The patient in focus, a 60-year-old female ex-smoker with a background of severe emphysema and COPD as well as a recent diagnosis of invasive poorly-differentiated adenocarcinoma of the colon, presented a unique diagnostic challenge. The lesion was in a very peripheral location, surrounded by large emphysematous blebs posing a high pneumothorax risk, which required sophisticated diagnostic methodologies. This case demonstrates a cutting-edge combination of interventional pulmonology techniques, including RAB with the use of balloon bronchoplasty, R-EBUS, ROSE, and CBCT with augmented fluoroscopy.

A recent meta-analysis has shown that RAB systems significantly increase diagnostic yield [7], and our case is consistent with these findings, demonstrating how a combined approach heightens the precision of tool placement, improves the accuracy of sample collection, and reduces the potential for complications such as pneumothorax while obtaining transbronchial biopsies of high-risk peripheral lung nodules. Traditional bronchoscopy techniques using thin scopes with R-EBUS or other catheter-based EMN strategies might have been unsuitable or even dangerous due to the patient's significant emphysema [8,9]. The novel technique, as illustrated by this case, permitted definitive diagnosis and management without inducing procedural pneumothorax, an achievement scarcely documented due to limited accessibility [10,11].

The complexity of this state-of-the-art interventional pulmonary procedure stems from the use of several concurrent techniques. These included RAB with electromagnetic navigation and R-EBUS confirmation of an eccentric view of the lesion, balloon bronchoplasty for closer navigation to the lesion, augmented fluoroscopy with 3D rendering for pinpoint diagnosis, and the strategic placement of gold fiducial markers to facilitate possible future stereotactic body radiation therapy for the treatment of the lesion. Together, these approaches highlight substantial progress in interventional pulmonology concerning the final diagnosis and management of PPLs, especially in higher-risk situations [10]. More importantly, they provide the pathway for appropriate and timely treatment without delays that might have arisen from potential post-procedural complications.

Our case further underscores the findings from the BENznidazole Evaluation For Interrupting Trypanosomiasis (BENEFIT) trial conducted by Chen et al. [12]. This pioneering study was the first prospective, multicenter evaluation of RAB for patients with PPLs ranging from 1 cm to 5 cm. The trial assessed the safety and feasibility of the procedure, which utilized a novel RAB system. With R-EBUS for lesion localization and a comprehensive approach to transbronchial needle aspiration, complemented by ROSE, the study showcased a high success rate. Out of 54 patients analyzed, lesion localization was successful in 96.2% of cases, with pneumothorax occurring in just 3.7%, of which only half required tube thoracostomy. These outcomes indicate that RAB offers a high degree of accuracy and safety, in line with conventional procedures, but with potentially increased precision and reduced risk of complications [12-14].

In another study by Cumbo-Nacheli et al., 20 patients who had suspected malignancy underwent RAB with CBCT and 100% confirmed tool-in-lesion before sampling [15]. Forty percent of the lesions were peripheral in Cumbo-Nacheli’s study, and there was an overall sensitivity for malignancy of 86.6%. The findings of the aforementioned investigation are all consistent with our case report, all suggestive of a strong foundation for considering RAB as a standard of care for the diagnosis of high-risk PPLs, though larger prospective studies are warranted.

RAB has expanded the horizons of diagnostic precision, with biopsy yields reported to lie between 69.1% and 81.7% [14,16]. In this realm, intraprocedural CBCT imaging is a critical tool to assess or confirm the placement of biopsy devices. Pritchett et al. evaluated the safety and diagnostic yield (DY) of combining intraprocedural CBCT data with augmented fluoroscopy during catheter-based electromagnetic navigation bronchoscopy (ENB) guided biopsy of PPLs [4]. Their retrospective data encompassed 93 lesions in 75 patients, revealing an overall DY of 83.7% (95% CI, 74.8%-89.9%) [4]. The analysis suggested that DY was not significantly impacted by the lesion’s size, location, visibility under standard fluoroscopy, or the presence of a bronchus sign. Pneumothorax was a rare complication, occurring in only 4% of the cases. The study credited the high DY to the synergistic use of CBCT with augmented fluoroscopy in ENB.

It is important to recognize that smaller peripheral lesions are less inclined to exhibit a bronchus sign [17], an anatomical marker that facilitates lesion navigation, minimizes the extent of lung parenchyma traversal, and improves accessibility even when using tools with limited flexibility [18]. In our case, the lesion's lack of a bronchus sign introduced additional procedural intricacies requiring balloon bronchoplasty to navigate closer to the lesion. Nonetheless, using CBCT-guided endobronchial biopsy in conjunction with augmented fluoroscopy enabled us to navigate these complexities successfully, illustrating the potential of these technologies to address the challenges presented by PPLs without a bronchus sign [19].

The importance of such an integrated approach becomes even more critical considering that lung cancer continues to be the predominant cause of cancer-related deaths globally, and the majority of lung nodules are in the periphery, as seen during the NELSON (Nederlands-Leuvens Longkanker Screenings Onderzoek) trial [20], underscoring the need for enhanced diagnostic precision provided by an integrated approach.

## Conclusions

This case report highlights the crucial impact of advanced interventional pulmonology technologies, particularly the integration of robotic bronchoscopy with CBCT-guided endobronchial biopsy and augmented fluoroscopy, for the accurate diagnosis and effective management of peripherally located high-risk pulmonary nodules.
